# Situation of the Elderly Living Alone: Morbidity and Services Provided from the Field of Primary Health Care of Gran Canaria

**DOI:** 10.3390/healthcare10101861

**Published:** 2022-09-24

**Authors:** Candelaria de la Merced Díaz-González, Lidia Esther Nuez-Herrera, Milagros de la Rosa-Hormiga, Antonio Cabeza-Mora, Josué Gutiérrez-Barroso

**Affiliations:** 1 Department of Nursing, University of Las Palmas de Gran Canaria, Juan de Quesada, 30, 35001, 35016 Las Palmas de Gran Canaria, Spain; 2 Primary Care Management of the Health Area of Gran Canaria, Calle Luis Doreste Silva, 36-44, 35006 Las Palmas de Gran Canaria, Spain; 3 Department of Sociology and Anthropology, University of La Laguna, Calle Padre Herrera, s/n, 38200 La Laguna, Spain

**Keywords:** Adjusted Morbidity Groups, elderly adults, primary health care

## Abstract

The elderly suffer a greater number of health problems and have greater need for assistance and care. (1) Background: to determine the profile of the elderly who live alone, identified according to the Primary Care Health Record of Gran Canaria, and to analyze the sociodemographic data of the target population and determine the characteristics related to morbidity. (2) Methods: descriptive, prospective, cross-sectional study carried out in the Primary Health Care Management of Gran Canaria. The study population was all adults over 65 years of age living alone. The instrument used was the Drago-Electronic Health Record. Data analysis was carried out using RStudio version 1.1.447 software, and descriptive analysis and inferential analysis were carried out using the Chi-square values, T-test for independent samples, and ANOVA. (3) Results: The sample amounted to 8679 subjects, predominantly female sex (86.14%) and with a mean age of 79.4 years. Of the sample, 6.4% lived alone. Based on the classification by Adjusted Morbidity Groups (AMG), subjects with “moderate complications” predominated at 45.5%. (4) Conclusions: It is necessary to implement this type of stratification tool, which allows interventions to be carried out in elderly people at risk.

## 1. Introduction

The increase in life expectancy at a global level is associated with growth in the older adult population (2020) [1]. According to data from the Spanish National Institute of Statistics (INE) (2020) [2], 19.6% of the population is over 65 years old, with expectations that by 2035 it will reach 26.5% and by 2050 31.4%, and with those over 100 years of age going from 12,551 today to 217,344 by the year 2070. A higher percentage of women is maintained compared to men, and differences in life expectancy continue, with women reaching 90 years on average by 2069, compared to men reaching an average of 85.61 years. In another report (2020) [3], it is shown that in 2020, there were more than 1,944,000 single-parent households (10.37% of the total), highlighting those made up only of women at 1,582,000 households (81.3%). If we focus on people over 65 years of age, it occurs again, with 515,400 (81.4%) single-parent households of women over 65 years of age, compared to 123,700 (18.6%) men. The widower marital status stands out as the most frequent in both sexes, with 435,000 (84.4%) women compared to 90,000 (72.7%) men.

The Autonomous Community of the Canary Islands [3], where this study was carried out, is the community that will see the third-highest population growth within the next 13 years, of 8.4%, increasing the population to 607,000 older people (OP), which would mean 25% of the total population (compared to the current 16.6%). All of the above suggests that in a short time the Canary Islands will have a high number of OP, which will generate an increase in the number of OP living alone.

It is expected that by 2033 (2020) [4] almost 29% of Spanish households will be made up of a single person, in addition to maintaining the growing trend and the single-person modality. In the case of the Canary Islands, it will occupy seventh place at the national level, with slight differences between the two provinces, where the province of Las Palmas de Gran Canaria would account for 51%.

### Brief Statement of the Problem: Morbidity and Services Provided

It is known that the aging trend in the Canary Islands is on the rise and that it is an inevitable phenomenon. This increase will come at the cost of an increase in chronic pathologies, due in part to the lifestyles of the Canary Islander population (2015) [5], with a direct impact on the presence of comorbidity and consumption of health resources [6], generating greater complexity in the management of this population group. All of the above leads to a growing demand for care by social and health professionals who will have to adapt and distribute resources efficiently.

Morbidity and multimorbidity (MM) is on the rise, as confirmed by a study carried out in 10 European countries between 2007 and 2015 [7], where there was an increase in the latter in all the countries studied, and where Spain reached 39.9% in persons over 50 years old—better than other countries in Europe, where Belgium and Italy obtained the worst results (47.5% vs. 46.1%, respectively), and Switzerland with the best results at 26.2%. In some documents it is indicated that morbidity in Spain is 65%; if we go to the report of the Ministry of Health [8], morbidity in the Autonomous Community of the Canary Islands (CAC) is 75.6% and MM 54.6%.

In Spain in 2016, the stratification of the population in the SNS project was developed, with the stratification tool by Adjusted Morbidity Groups (GMA^®^) [9] implemented in 13 autonomous communities, allowing classification based on morbidity and complexity of almost 38 million inhabitants at the end of 2015 (Ministry 2018). The classification was made based on morbidity and five levels (N) of complexity, wherein the higher the number, the higher the complexity. The assigned colors indicate the following (based on percentile of complexity weight over the general population): gray color = <80 without any chronic pathology; vanilla = <80 with some chronic pathology; yellow = >80 and <95; orange = >95 and <99; red = >99 (Ministry 2020). The results refer to the population over 14 years of age, and in the CAC [10] the vanilla color predominated.

The Primary Care Management of Gran Canaria (PCMGC) includes the Adjusted Morbidity Groups (AMG) in the Drago Primary Care Electronic Medical Record (Drago-EHRPC). The AMG allows the stratification of the population for clinical-care management purposes, as well as the appropriate distribution of resources based on the specific needs of each identified population group [9]. Therefore, the AMG is a good predictor of resource consumption and health-care needs. The question that then arises is that, although there are tools available that point to where to orient efforts in OP care in the PC setting, there is concern about OP living alone and the possible consequences that loneliness may have on their physical-health status, psychology, and their social sphere [9], or even mortality [11].

The population included in the PHC Health Card is stratified into five AMG groups (based on multimorbidity and complexity), where each stratum/group is identified by colors from lower to higher complexity, ranging from gray to red (through vanilla, yellow, and orange). Gray represents low-complexity, vanilla low–moderate-, yellow moderate-, orange moderate–high-, and red high-complexity patients.

Population aging is a reality that cannot be questioned, as well as an achievement and sign of success of the population. Spain has one of the highest rates of aging in Europe, standing at 125.6 in 2020 [12] and continuing to rise. In addition, the trend towards an aging population is associated with multiple pathologies, chronicity, dependency, and loneliness, among others, highlighting the need to carry out studies in this population group, with the objective of detecting problems in advance through the use of tools like AMG. Identifying the OP who live alone in PCMGC, their characteristics, and their degree of morbidity is of vital importance to identify needs, the functioning of AMG within the Drago-EHRPC, and the results that are useful to managers to plan new action strategies in view of the similar growth of OP in the Autonomous Community of the Canary Islands by 2035. In the Canary Islands no studies have been carried out on Primary Care Management.

The aim of this study is to understand the profile of the elderly living alone, identified according to the DEHRPC registry in Gran Canaria; to analyze the sociodemographic data of the target population (age, sex, Basic Health Area); and to determine the characteristics related to morbidity.

## 2. Materials and Methods

This is a retrospective, cross-sectional, descriptive study carried out in the community setting in the elderly population of individuals aged 65 or older of the Primary Care Management of Gran Canaria (PCMGC), Drago Electronic Health Records of Primary Care (Drago-EHRPC). Drago-EHRPC was accessed from 2 June 2020 to 2 September 2020, and among the population over 65 years of age (134,191), those living alone were identified based on an assessment of Marjory Gordon’s patterns (according to pattern 8) in the period from 1 June 2018 to 1 June 2020.

The variables included were age, sex, hospital morbidity group (and its percentile and associated color in Drago-EHRPC), presence of the caregiver, whether they presented immobilization, the number of consultations in the different primary-care specialties, and the Basic Health Areas (BHAs) to which they were assigned.

Inclusion criteria: older people aged 65 years or older enrolled in the PCMGC during the period under study.

Data analysis: regarding data analysis, Rstudio^®^ software, version 4.0.2 (Ross Ihaka and Robert Genleman in Auckland, New Zealand) was used. In addition, descriptive analysis and inferential analysis were performed using the Chi-square values, T-test for independent samples, and ANOVA, as appropriate.

## 3. Results

This section shows the results obtained from the exploitation of the database on the care of 65-year-olds living alone who were included in the Drago Electronic Health Records of Primary Care of Gran Canaria (Drago-EHRPC).

Thus, after filtering the data, the sample amounted to 8679 subjects, which represents 6.4% of the population over 65 years of age included in the Drago-EHRPC.

As can be seen in Table 1, there were more women than men living alone (women 86.14%, men 13.86%). Furthermore, by age, the age range was between 65 and 104 years, with the mean being 79.4 years with a standard deviation of 7.63.

This section is divided by subheadings. It should provide a concise and precise description of the experimental results, the interpretation of them, and the experimental conclusions that can be drawn.

The relationship of the people in the database based on the Basic Health Areas (BHA) who presented a greater weight in the sample was as follows: Alcaravaneras (6.24%), Guanarteme (5.36%), Escaleritas (5.16%), San Gregorio (5.05%), Puerto (4.98%), Doctoral-Vecindario (4.93%), and Miller Bajo (4.77%), all urban areas.

The percentage of people who live alone and have a caregiver assigned to them was 27.6%. Finally, to conclude with the sociodemographic profile, it should be noted that 16.92% of people over 65 years of age who live alone were immobilized.

The Adjusted Morbidity Groups (AMG) were analyzed, and the values associated with them can be found in Table 2, as well as the percentiles of this variable. Thus, the mean of the AMG was 18.80, with a standard deviation (SD) of 9.79. These values, together with the fact that their range was between 0 and 75, led us to think that the mean is not very representative since the extreme values significantly altered the average. This issue was observed when analyzing the percentiles: The 100th percentile had an AMG value of 75; in addition, the median of the percentiles was 92, which allows us to state that the values were highly concentrated around the median and mean values, and there were a few values at the extremes (especially around the minimum values) that altered the average values.

When analyzing the AMG by color according to Drago-EHRPC, it was observed that most of them were assigned a yellow color denoting moderate complexity (45.54%), followed by orange (moderate–high, 33.02%), vanilla (moderate, 17.05%), red (high, 4.31%), and gray (very low, 0.08%). The data shown in Table 3 are based on these colors and the immobility situation.

To evaluate the ratio of care provided to the PCs, we extracted data related to the number of consultations for the different specialties (Table 4), with the highest mean number of consultations being those related to medicine (9.9), followed by nursing (7.82). In both types of consultation, there was a large dispersion when the standard deviation was observed, which only indicates that there were some extreme values (especially in PCs with a high demand for consultations, with the maximum values of consultations seen in Medicine and Nursing) that affected the average. Thus, it can be stated that 75% of people used less than 13 consultations in medicine and 9 consultations in nursing, whereas others used them up to 102 and 346 times, respectively.

On the other hand, the number of consultations in the rest of the specialties listed in the table was considerably lower, as they were used by only a few people. As an example of this, it can be seen that in Liaison Nursing, Social Work, General Emergency, and General Incident, 75% of the sample never visited any of them.

Next, we analyzed the bivariate crosses between the quantitative variables based on sex, thus applying a sex perspective to the research. Table 5 shows that women over 65 years of age who live alone were significantly older than men, with significant differences when Cohen’s D was observed.

When analyzing the consultations made in primary care according to the sex of the person over 65 years of age (Table 6), it can be seen that there were significant differences in all the variables except in Liaison Nursing, General Emergency, and General Incident, perhaps because this is where there were the fewest cases, as seen above.

Thus, women attended significantly more medical consultations than men, whereas men attend more nursing and social-work consultations.

Analyzing the AMG, it was observed that there were significant differences (α < 0.01) between men and women in the variables related to it (Table 7). Thus, men in general had a higher AMG than women.

Table 8 also shows significant differences in the color of the triangle of the AMG associated with DEHRPC. Thus, there were significant differences between men and women in all categories except the orange one, where the values were very similar. Considering the data, in both sexes the colors yellow, orange, and vanilla were most frequent.

Regarding whether the person had a caregiver or was immobilized, based on the sex (Table 9), the percentage of women who had a caregiver was significantly higher than that of men, as was the case with immobilization.

After cross-referencing the variables based on the sex of the users, we cross-referenced all the variables, taking into account the color associated with the patient’s AMG in Drago-EHRPC. This allowed us to associate morbidity with consultations and other sociodemographic variables.

Based on age (Table 10), there were significant differences in the AMG colors. Thus, the mean age was significantly higher in the orange and red colors, whereas it was slightly lower in yellow, gray, and vanilla.

Taking into account the number of consultations according to AMG color (Table 11), it can be seen that there were significant differences in all types of consultations except for Liaison Nursing and General Incident, although the differences in General Emergency consultations were low, despite being statistically significant.

In general, in medical consultations, the highest averages corresponded to the red and orange AMG colors, as was the case in Nursing and Social Work. It is worth highlighting the medical consultations, since the AMG color explained between 19 and 22% of this variable, which denotes significant explanatory power.

To conclude with the crosstabs of the variables based on the color of the AMG associated with the patient in Drago-EHRPC, Table 12 shows the analysis of the AMG values based on the associated color “weight” (the variable used to evaluate the predictive capacity was the complexity weight obtained by the respective stratifiers). Thus, the AMG values were higher in the red and orange colors, which is in line with what was analyzed in Table 10: The associated high AMG values refer to the red and orange colors. Thus, the associated color variable in Drago-EHRPC explained between 85 and 86% of the AMG, indicating that these variables are closely related.

**Figure 1:**
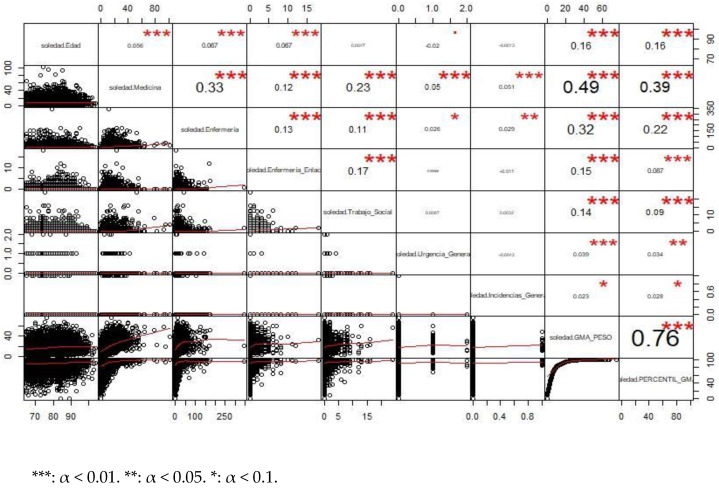
Correlations between all quantitative variables. Basic Health Areas of Gran Canaria. 2018–2020. Correlation coefficients and scatter plots.

Figure 1 shows a bivariate analysis of the quantitative variables in the database through the display of correlation coefficients and scatter plots.

Except for the correlation between the AMG and its percentile (which measured something very similar), we found that there were significant differences. Thus, the variable that correlated most with medical consultations was the AMG variable and nursing consultations. Thus, the more consultations you had in medicine, the more consultations you had in nursing and the higher the AMG value you had. This can be intuited from Table 10, which is related to AMG. The same was true for nursing consultations, which had a highly positive correlation coefficient associated with the AMG variable.

**Figure 2:**
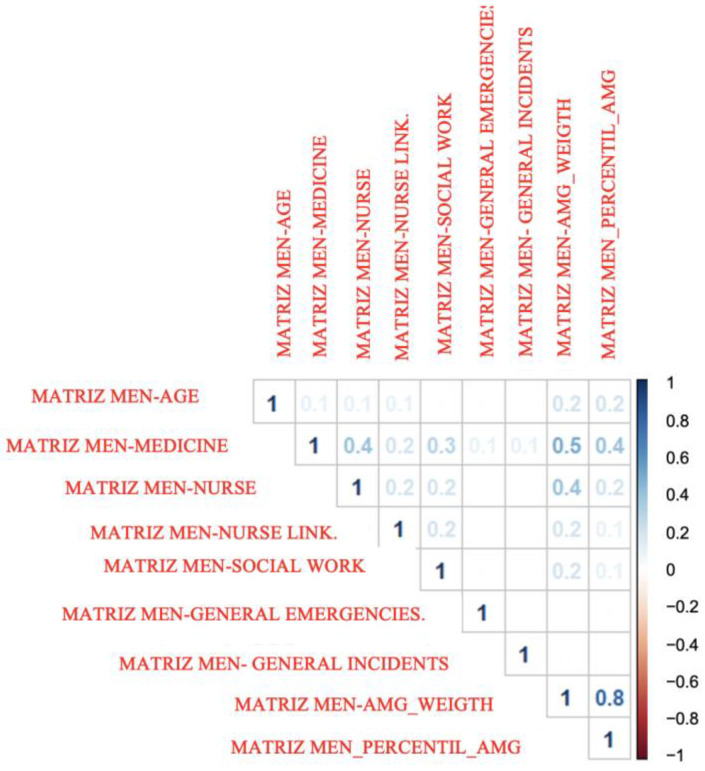
Correlations between all quantitative variables for men. Basic Health Areas of Gran Canaria. 2018–2020. Correlation coefficient.

**Figure 3:**
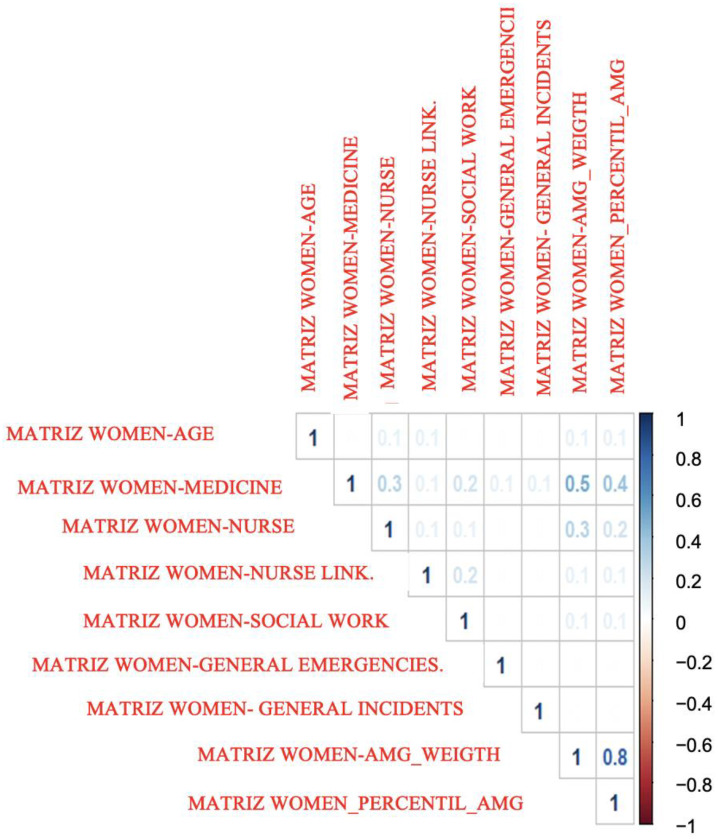
Correlations between all quantitative variables for women. Basic Health Areas of Gran Canaria. 2018–2020. Correlation coefficient.

If we analyze the previous correlations differentiating the group of men and women, we obtain Figure 2 (correlations for the group of men) and Figure 3 (correlations for the group of women). As can be seen, the differences between the values of both are very small.

## 4. Discussion

The reasons for older people (OP) living alone are the result of a combination of different types of arguments, each case having singularity. It can be affirmed that there are two main reasons for living alone: (a) personal reasons, which show that they are willing to remain at home (due to attachment to their home, proximity to their children, permanence in the social context, freedom in the design of their daily activities, economic self-sufficiency, or the serenity of the home), or (b) circumstantial obligation, due to the characteristics or situation of the family environment (lack of descendants, shortage of space in the homes of relatives, feeling of emptiness in someone else’s home, the deep-rooted idea of being a nuisance, not wanting to deteriorate family relations) [13].

The percentage of women living alone in Gran Canaria exceeds 86%, compared to only 13% in the case of men. These data are higher than the reality at the national level (2020) [14], where women account for 72.3% of single-person households made up of 65-year-olds. This pattern is repeated in OP 85 years of age, where women double (43.2%) men (21.8%). It is to be expected that these data will increase, and that the Canary Islands, Spain, will approach the data of other European countries, where old age increases the probability of living alone [6].

We detected that women older than 65 years who live alone are significantly older than men. These data resemble those reported by Pin et al. [15]; in addition, they found an association between being a man living alone and increased mortality. In addition, the higher life expectancy in women (85.8 years for women vs. 80.5 years for men) implies a higher probability of being widowed (2020) [14], data that are reflected in the national statistics [3], where 84.4% of women are widows.

Concerning the OP living alone based on the BHA to which they are attached, the percentages showed urban areas with a higher percentage of OP living alone, compared to the BHA of Tejeda–Artenara (0.25%), with a low percentage. The reason could be due to the disruption of family relationships in urban versus rural areas. Rural communities are attributed with a denser social fabric, formed by more traditional and closer social relationships, with richer and more effective mutual-support networks, i.e., greater social capital [16]. In addition, previous studies [17] have found that one third of the OP in urban areas are at risk of social isolation and feel lonely.

After identifying that 72.4% of the OP who live alone do not have a caregiver, our concern arose to understanding the specific situation within the AMG of these subjects, which could shed light on the percentage within this candidate group that requires further follow-up; 4.8% were in a situation of high morbidity, and 43% moderate–high, which would indicate that almost 48% (adding both) could be a subsidiary of greater follow-up. Although no data have been found to compare with the group without a caregiver, nor in general in OP, the increase in life expectancy together with the increasing morbidity (65% vs. 75.6%) and MM in Spain and CAC (39.9% vs. 54.6%) [7,8] could suggest an increase in higher levels of AMG. These data show a great difference between the general and local data, always bearing in mind the inclusion of the general population vs. the population > 14 years of age.

Although in our study the most frequent morbidities were not addressed due to the high percentage of them in CAC, it is necessary to comment that among the most frequent pathologies are sensory alterations (61–63%), musculoskeletal conditions (54–56%), and hypertension (32–37%) [18]. In addition, the presence of multimorbidity (MM) is the norm and not the exception [19], being the population with MM the one that presents worse results with respect to the quality-of-life results and high consumption of socio-health resources.

This could indicate the need to generate a parallel digital tool for moderate–high morbidity groups that require more frequent follow-up with more specific interventions.

The colors of the Adjusted Morbidity Groups (AMG) triangle showed significant differences between men and women (<0.01), with the latter being less likely to be affected. Men have a lower life expectancy and present higher morbidity, which may be the cause of complications such as mortality and death occurring earlier than in women. It was also found that the mean age was significantly higher in the orange and red colors, whereas it was slightly lower in yellow, gray, and vanilla, which may be related to the increase in age and the increase in complications. Barrios Cortés et al. [20] showed that high risk was associated with greater age, male sex, immobility, and greater number of chronic diseases, among others. In addition, significant differences were found between men and women in all AMG categories except the orange one, where the values were very similar. Considering the data, men figured to a greater extent in the colors vanilla, red, and gray, whereas women figured to a greater extent in the yellow color (moderate complication). Data at the national level do not specifically show studies on AMG in OP who live alone, and neither do specific studies on OP. In addition, in some cases three risk levels are used in the classification of AMG [20], aspects that make comparison difficult. According to the existing data on CAC, the results refer to the population over 14 years of age [10]: gray—58,036 (3.6%) subjects, vanilla—225,954, yellow—61,035, orange—19,694, and red—2220. The document does not present the percentages for each color; therefore, based on the sample (1.7 million and knowing that 75% have chronic disease), the percentages per color would be 38.6%, 17.8%, 4.8%, 1.5%, and 0.17%, respectively. Obviously, these data are isolated from our results and the profile of the study population differed a lot; thus, the orange and red colors (moderate–high) showed a scarce 1.67% compared to ours, at 37.3%. Studies with similar characteristics are needed.

Regarding the presence of a caregiver or immobilization based on the sex of the person, the percentage of women who have a caregiver was significantly higher in women than in men, as was the case with immobilization. This may be because women were older on average and had greater caregiver and immobilization needs. In the old age of the population there was a feminization [6,21], which implies the birth of more men than women. From the age of 50 this difference stabilized, and the more advanced the age the greater the feminization. Thus, it is prudent to affirm that, the longer one lives, the greater the probability of pathologies, functional deterioration, and immobilization, requiring at some point support from caregivers due to the total or partial loss of autonomy.

Medical consultations had the highest means, corresponding to the AMG colors red and orange, as did Nursing and Social Work. This may indicate that the AMG colors associated with each patient in Drago-EHRPC discriminate very well in relation to the number of consultations that patients tend to use, since a higher AMG indicates greater morbidity, greater complexity, and greater demand for social and health services. These data coincide with the results of Barrio-Cortes et al. [22], where contact with the physician was higher compared to nursing, and with higher utilization based on female sex, age, and clinical need (high risk of morbidity). Cassell et al. [23] showed in their study a strong association between MM and the configured annual rate of GP consultations, where patients with MM had 2.56 times more consultations than patients without MM; in addition, the latter was associated with female sex, increased age, and lower economic resources, a parameter not valued in our study.

A total of 75% of the sample of OP who live alone made a low demand for consultation services, despite the fact that after adding the OP included in the yellow color for moderate complexity (45.54%) and orange for moderate–high complexity (33.02%) their sum exceeded 75%, a fact that makes us doubt whether they really do not go to consultations, or on the contrary whether they go to private health services. As we do not have these data, it would be necessary to obtain socio-economic information that alerts us before the actual abandonment of follow-up of their pathologies occurs and, consequently, adherence to treatment. As is seen later, some authors [24] have stated the need to include additional data in the AMG.

The bivariate analysis (Figure 1) showed a positive correlation between the AMG variable and physician consultations and nursing consultations. Thus, the more consultations in medicine, the more consultations in nursing and the higher the AMG value. The higher the AMG, the greater the complexity and the greater the demand for social and health services, especially medical and nursing health services, because they are poly-medicated subjects with needs for follow-up and treatment adjustments and periodic control of pathologies, among others, where the greater the morbidity, the greater the demand for health care services—data similar to those discovered by Fulmer et al. [25] in their proposal for providing better health and medical care for older adults.

Differences in the use of medical and nursing consultations were discovered, with women attending more medical consultations and men attending more nursing/social-work consultations, data that coincide with the results of Barrio-Cortes et al. [22]. When we consulted the European Health Survey in Spain (2020) [20], it coincided with these data; however, when we narrowed it down by age group to between 75–84 years and 85 years, percentages of over 40% for both sexes attended the family doctor’s office in the previous four weeks [26].

The data showed that 6.4% of OP 65 years old or older live alone. There are no uniform conclusions that living alone is associated with increased mortality, as some studies have reported no increase in mortality [27] or a decrease in mortality [28]. It is important to differentiate between the experience of “living alone” where it is a voluntary decision and the feeling of “loneliness” involuntary decision, on the latter there are a greater number of studies that state that it is a factor associated with mortality [29,30], and this effect is slightly stronger in men [30].

Although aspects such as health spending or socioeconomic level in this age group (not included in this AMG) were not mentioned previously, both are related to MM; thus, high levels of AMG generate a high social health cost per the high demand for services, as stated by Cassell et al. [23]. This aspect should be mentioned, because one of the objectives of the AMG is to reduce health spending and distribute resources more equitably.

In the present study, the focus was on the AMG included in PCMGC, but it would be useful to be able to access this tool in the hospital setting, or a tool developed jointly. It is necessary to reflect that the AMG is not the only staging tool used in Spain; it is also possible to find the Adjusted Clinical Groups (ACG) and the Clinical Risk Group (CRG^®^). Arias-Lopez et al. [24] carried out a comparative analysis at the level of these three stratification tools in three Spanish autonomous communities, showing that the GMA are good predictors of almost all the variables; however, they did not include additional data such as demographic data or prescriptions (ATC codes), which is included by the ACG^®^ and the CRG^®^. It would also be very useful to develop a predictive logistic-regression model involving both primary and hospital care, as carried out by Jones et al. [31], where the combination of hospitalization variables and the evaluation of the community physician can easily predict the risk of readmission.

We support the proposals of other authors who affirm that it is a flexible tool that can be adapted and recalculated in other organizations [32], and that it can also improve the predictive capacity for hospital admission by adding other dimensions [24]. The development of its own groups would allow each organization to have a tool adapted to the population and its needs, especially in care programs for chronic patients, but this requires more studies and financial investment.

However, there are studies that advocate the implementation of AMG (in Spain it can only be found in some communities), and the Spanish Ministry of Health is considering the possibility of including this stratification tool in the National Health System.

## 5. Conclusions

A total of 6.4% of the OP studied live alone, with the majority being females (86%) who were significantly older than males. Regarding the demand for consultations, medicine and nursing were the most requested, with a scant 25% of the sample, with women attending significantly more medical consultations than men, whereas men attended more nursing and social-work consultations. Regarding the remaining 75% of OP who live alone, they made a very low demand for consultations, and could have been going to private health services. There were significant differences between levels of AMG and sex, where men had higher averages, as well as between age and AMG colors, with the subjects with the highest average age being those located in the highest levels (red and orange). The demand for consultations and the AMG color also presented significant differences, where the colors red and orange (greater comorbidity) demanded a greater number of consultations. The associated color variable in Drago-EHRPC explained between 85 and 86% of the AMG, indicating that these variables are closely related. The use of health services by patients with a high level of risk assigned by the AMG was higher for medical and nursing consultations. Predisposing factors included female sex and age. The AMG colors associated with each patient in Drago-EHRPC discriminated very well in relation to the number of consultations that patients usually made, since a higher AMG indicates greater morbidity, greater complexity, and greater demand for social and health services.

It would be pertinent for this tool to be reviewed and to include other indicators that were not present at the time of data collection, such as economic resources, accompaniment (no caregiver, which is included), and the presence of private health insurance, among others, data that could be useful to correlate with morbidity levels. In addition, the clear increase in life expectancy, the MM, the tendency for more OP to live alone, and the few studies in Spain focused on morbidity and AMG in this age group in single-person households make it necessary to create new lines of work, due to the difficulty in finding data to compare.

It can be concluded that AMG are a very useful tool as a measure of morbidity and can help in the collection, exploration, and analysis of information in the health environment, improving the adjustment of indicators and the development of predictive models (e.g., the risk of hospital readmission); however, each National Health System should implement this type of staging tool, including new indicators based on the characteristics of its population, which allows for intervention in risk groups and the prevention of complications, chronicity, limitations, and, therefore, increased health costs. However, it is necessary for each institution to update its tool and adapt it to the new indicators that may be capable of being incorporated.

## 6. Limits

Among the limitations of the study is the design chosen, since the nature of the associations cannot be interpreted in terms of cause–effect. In addition, the possibility of the existence of biases in the information recorded in Drago-EHRPC should be taken into account. 

## Figures and Tables

**Table 1:** Persons over 65 years of age living alone based on sex and age. Basic Health Areas of Gran Canaria. 2018–2020. Medians and percentages.

Age	Sex
Min.: 65.00	Male: 13.86%
1st Qu.: 73.00	Female: 86.14%
Median: 80.00	
Mean: 79.40	
Sd: 7.63	
3rd Qu.: 85.00	
Max.: 104.00	

**Table 2:** Variables of the Adjusted Morbidity Groups (AMG) of persons over 65 years of age living alone. Basic Health Areas of Gran Canaria. 2018–2020. Descriptive.

AMG	Percentil_AMG
Min.: 0.00	Min.: 0.00
1st Qu.: 12.00	1st Qu.: 84.00
Median: 17.00	Median: 92.00
Mean: 18.80	Mean: 88.19
Sd: 9.79	Sd: 11.90
3rd Qu.: 24.00	3rd Qu.: 96.00
Max.: 75.00	Max.: 100.00

**Table 3:** Persons over 65 years of age living alone with some type of immobilization according to the color of the patient’s AMG triangle in Drago. Basic Health Areas of Gran Canaria. 2018–2020. (Percentages—%).

	Inmovilized	
	No.	Sí
Gray	0.08%	0.07% ***
Vanilla	18.72% ***	8.86% ***
Yellow	47.35% ***	36.65% ***
Orange	30.67% ***	44.55% ***
Red	3.18% ***	9.88% ***

***: α < 0.01.

**Table 4:** Consultations made in primary care for people over 65 years of age who live alone, based on different specialties. Basic Health Areas of Gran Canaria. 2018–2020. Descriptive.

Medicine	Nursing	Nursing_Link	Social Work	General Emergency	General_ Incident
Min.: 0.00	Min.: 0.00	Min.: 0.00	1st Qu.: 0.00	Min.: 0.00	Min.: 0.00
1st Qu.: 5.00	1st Qu.: 1.00	1st Qu.: 0.00	Median: 0.00	1st Qu.: 0.00	1st Qu.: 0.00
Median: 8.00	Median: 4.00	Median: 0.00	Median: 0.00	Median: 0.00	Median: 0.00
Mean: 9.90	Mean: 7.82	Mean: 0.13	Mean: 0.27	Mean: 0.01	Mean: 0.01
Sd: 7.73	Sd: 13.24	Sd: 0.65	Sd: 1.02	Sd: 0.09	Sd: 0.05
3rd Qu.: 13.00	3rd Qu.: 9.00	3rd Qu.: 0.00	3rd Qu.: 0.00	3rd Qu.: 0.00	3rd Qu.: 0.00
Max.: 102.00	Max.: 346.00	Max.: 18.00	Max.: 24.00	Max.: 2.00	Max.: 1.00

**Table 5:** Age of persons over 65 years old living alone based on sex. Basic Health Areas of Gran Canaria. 2018–2020. Medians.

	Male	Female	Cohen’s D
Age	77.47 ***	80.19 ***	0.36

***: α < 0.01.

**Table 6:** Consultations made in primary care for persons over 65 years of age living alone based on the different specialties and sex. Basic Health Areas of Gran Canaria. 2018–2020. Averages.

	Male	Female	Cohen’s D
Medicine	9.09 ***	10.24 ***	0.15
Nursing	8.82 ***	7.41 ***	0.11
Nursing_Link	0.14	0.13	---
Social Work	0.31 **	0.25 **	0.05
General_Emergency	0.01	0.01	---
General_Incident	0.00	0.00	---

***: α < 0.01. **: α < 0.05.

**Table 7:** Adjusted Morbidity Group (AMG) variables of persons over 65 years of age living alone based on sex. Basic Health Areas of Gran Canaria. 2018–2020. Medians.

	Male	Female	Cohen’s D
AMG_Weight	19.33 ***	18.58 ***	0.08

***: α < 0.01.

**Table 8:** Colors of the AMG triangle of persons over 65 years of age living alone based on sex. Basic Health Areas of Gran Canaria. 2018–2020. (Percentages—%).

	Colors	Male	Female
Patient’s AMG Triangle Color in Drago	Gray	0.24% ***	0.02% ***
	Vanilla	19.52% ***	16.03% ***
	Yellow	40.46% ***	47.63% ***
	Orange	33.61%	32.78%
	Red	6.18% ***	3.54% ***

***: α < 0.01.

**Table 9:** Living situation and immobilization of persons over 65 years of age living alone according to sex. Basic Health Areas of Gran Canaria. 2018–2020. (Percentages—%).

		Male	Female
Caregiver	YES	23.04% ***	29.53% ***
	No	76.96% ***	70.47% ***
Inmobilized	No	86.38% ***	81.72% ***
	YES	13.62% ***	18.28% ***

***: α < 0.01.

**Table 10:** Age of persons over 65 years of age living alone based on the color of the patient AMG triangle in Drago-EHRPC. Basic Health Areas of Gran Canaria. 2018–2020. Medians.

	Gray	Vanilla	Yellow	Orage	Red	Interval Where the Power Is Located (%)
Age	76.00 ***	77.15 ***	79.10 ***	80.74 ***	81.12 ***	(2,1;3,4)

***: α < 0.01.

**Table 11:** Consultations performed in primary care for persons over 65 years of age living alone based on the color of the patient AMG triangle in Drago-EHRPC. Basic Health Areas of Gran Canaria. 2018–2020. Medians.

	Gray	Vanilla	Yellow	Orange	Red	Power (%)
Medicine	0.86 ***	4.99 ***	8.55 ***	13.08 ***	19.78 ***	(19,3;22,1)
Nursing	0.29 ***	3.32 ***	6.08 ***	10.74 ***	21.81 ***	(8,0;10,3)
Nursing_Link	0.00	0.05	0.08	0.19	0.50	---
Social Work	0.29 ***	0.12 ***	0.21 ***	0.37 ***	0.78 ***	(1,3;2,4)
General_Emergency	0.00 **	0.00 **	0.01 **	0.01 **	0.01 **	(0,0;0,3)
General_Incident	0.00	0.00	0.00 *	0.00	0.00	---

***: α < 0.01. **: α < 0.05. *: α < 0.1.

**Table 12:** Adjusted Morbidity Group (AMG) variables of persons over 65 years of age living alone according to the patient AMG triangle color in Drago-EHRPC. Basic Health Areas of Gran Canaria. 2018–2020. Medians.

	Gray	Vanilla	Orange	Yellow	Red	Power (%)
AMG_weight	0.29 ***	7.00 ***	26.89 ***	14.90 ***	45.04 ***	(85,3;86,1)

***: α < 0.01.

## Data Availability

The authors wish to thank the Primary Care Management of Gran Canaria—Canary Islands Health Service—for allowing access to the data. Without their authorization this project would not be a reality.
